# 1-Benzoyl­methyl-3-(2-thienylmeth­yl)-4-(2-thienylmethyl­eneamino)-1*H*-1,2,4-triazol-5(4*H*)-one

**DOI:** 10.1107/S160053681000005X

**Published:** 2010-01-09

**Authors:** Hasan Tanak, Metin Yavuz, Zeliha Lagap, Orhan Büyükgüngör

**Affiliations:** a Department of Physics, Faculty of Arts & Science, Ondokuz Mayıs University, TR-55139 Kurupelit-Samsun, Turkey; b Department of Chemistry, Karadeniz Technical University, Trabzon, Turkey

## Abstract

In the title compound, C_20_H_16_N_4_O_2_S_2_, one of the thio­phene rings is disordered [occupancy ratio 0.710 (4):0.290 (4)] and the disorder is of the flip type. An intra­molecular C—H⋯O hydrogen bond generates a six-membered ring with an *S*(6) motif.

## Related literature

For general background to 1,2,4-triazoles and thio­phenes, see: Santen (2003 ▶); Clemons *et al.* (2004 ▶); Chen *et al.* (1997 ▶); Mohareb *et al.* (2004 ▶); Collin *et al.* (2003 ▶). For the graph-set description of hydrogen bonds, see: Bernstein *et al. *(1995 ▶). For reference structural data, see: Allen *et al.* (1987 ▶). For related structures, see: Tanak *et al.* (2009 ▶); Akkurt *et al.* (2008 ▶); Ustabaş *et al.* (2009 ▶).
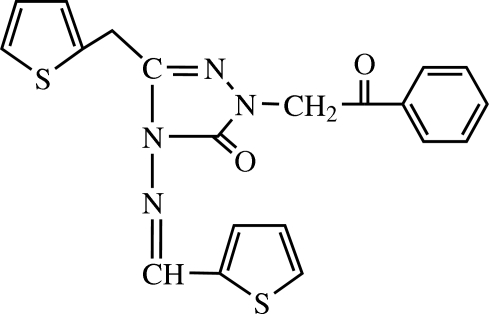


## Experimental

### 

#### Crystal data

C_20_H_16_N_4_O_2_S_2_
*M* *_r_* = 408.49
Monoclinic, 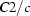

*a* = 25.287 (3) Å
*b* = 5.5347 (4) Å
*c* = 28.281 (2) Å
β = 102.430 (7)°
*V* = 3865.2 (6) Å^3^
*Z* = 8
Mo *K*α radiation
μ = 0.30 mm^−1^
*T* = 296 K
0.80 × 0.37 × 0.14 mm

#### Data collection

Stoe IPDS II diffractometer
Absorption correction: integration (*X-RED32*; Stoe & Cie, 2002 ▶) *T*_min_ = 0.776, *T*_max_ = 0.913
20676 measured reflections
3865 independent reflections
2676 reflections with *I* > 2σ(*I*)
*R*_int_ = 0.037

#### Refinement

*R*[*F* ^2^ > 2σ(*F* ^2^)] = 0.049
*wR*(*F* ^2^) = 0.141
*S* = 1.05
3865 reflections
272 parameters
104 restraints
H-atom parameters constrained
Δρ_max_ = 0.36 e Å^−3^
Δρ_min_ = −0.31 e Å^−3^

### 

Data collection: *X-AREA* (Stoe & Cie, 2002 ▶); cell refinement: *X-AREA*; data reduction: *X-RED32* (Stoe & Cie, 2002 ▶); program(s) used to solve structure: *SHELXS97* (Sheldrick, 2008 ▶); program(s) used to refine structure: *SHELXL97* (Sheldrick, 2008 ▶); molecular graphics: *ORTEP-3 for Windows* (Farrugia, 1997 ▶); software used to prepare material for publication: *WinGX* (Farrugia, 1999 ▶).

## Supplementary Material

Crystal structure: contains datablocks I, global. DOI: 10.1107/S160053681000005X/dn2520sup1.cif

Structure factors: contains datablocks I. DOI: 10.1107/S160053681000005X/dn2520Isup2.hkl

Additional supplementary materials:  crystallographic information; 3D view; checkCIF report

## Figures and Tables

**Table 1:** Hydrogen-bond geometry (Å, °)

*D*—H⋯*A*	*D*—H	H⋯*A*	*D*⋯*A*	*D*—H⋯*A*
C11—H11⋯O1	0.93	2.35	2.981 (3)	125

**Fig. 1.**
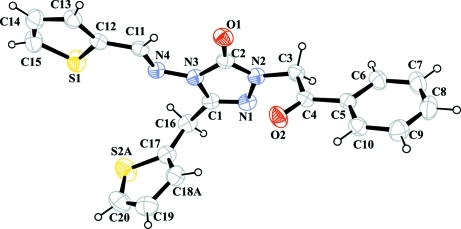
The molecular structure of the title compound, showing the atom-numbering scheme and 30% probability diplacement ellipsoids.

## supplementary crystallographic information

### Comment

1,2,4-triazole derivatives are known in the scientific literature for their wide pharmacological activity. Two main types of their activity are antiviral, antibacterial and antifungal activities, and central nervous system (*CNS*) activity. It was reported that compounds having triazole moieties such as Vorozole, Anastrozole and Letrozole appear to be very effective aromatase inhibitors very useful for preventing breast cancer (Ünver *et al.*, 2009; Santen, 2003; Clemons *et al.*, 2004). It is known that 1,2,4- triazol moieties interact strongly with heme iron, and aromatic substituents on the triazoles are very effective for interacting with the active site aromatase (Chen *et al.*, 1997). Over recent years, there has been an increasing interest in the chemistry of thiophenes because of their biological significance. Many of them have been widely investigated for therapeutic uses, especially as antifungal, antibacterial, antiinflammatory, anticonvulsant, antiasthmatic, and analgesic agents. They also were known to show anti-HIV, antiproliferative, germicidal, and D2 dopaminergic activities (Mohareb *et al.*, 2004). There are antimicrobial agents having different structures are frequently used in treatment of microbial infections. However, there is an increasing resistance to these drugs. Moreover, some of azole derivatives used as common antibiotics such as Amphotericin B posses a toxic effect on humans as well as their antimicrobial effects (Collin *et al.*, 2003). To overcome the development of drug resistance, it is crucial to synthesize a new class of antimicrobials possessing different chemical properties from those of used commonly.

The molecular structure of the title compound (I) is shown in Figure 1. Within the molecule of (I), a flip-disorder of the thiophene ring containing S2 is observed. There are two positions of the thiophene ring, rotated by *ca* 180° about the single C16—C17 bond. These two orientations are not equivalent; the site-occupation factors refined to 0.710 (4) and 0.290 (4). All the bond lengths and angles of (I) are within normal ranges (Allen *et al.*,1987; Akkurt *et al.*, 2008; Ustabaş *et al.*, 2009). In (I), thiophene rings and benzyl ring are bridged by 1,2,4-triazole ring system. The dihedral angles between the triazole ring A (N1/N2/C2/N3/C1), the benzyl ring B (C5—C10), the thiophene rings C (C12/C13/C14/C15/S1), D (C17/C21/C20/C19/S2), and E (C17/C18/C19/C20/S3) are 80.04 (14)° (A/B), 11.45 (15)° (A/C), 82.82 (17)° (A/D), 83.5 (2)° (A/E), 74.35 (15)° (B/C), 14.87 (17)° (B/D), 13.8 (2)° (B/E), 79.81 (17)° (C/D) and 80.2 (2)° (C/E). The torsion angles, (N3/N4/C11/C12) and (N2/C3/C4/C5) are 178.43 (19)° and 176.08 (19)°, shows that for the title compound, the side chain conformation induced by anti-conformations, respectively. The interatomic distances within the triazole ring of (I) are not equal. The C1—N1 is double bond and shorter than the conjugated C1—N3 and C2—N3 bonds. The molecular geometry of the triazole ring is in agreement values with the structure 4-(2,3-dihidroxybenzylideneamino)-5-methyl-2*H*-1,2,4-triazole-3(4*H*)-one (Tanak *et al.*, 2009).

The molecular structure is stabilized by C—H···O type hydrogen bond. An intramolecular hydrogen bond C11—H11···O1, forming rings with the graph set S(6) (Bernstein *et al.*, 1995), details of which are given in Table 1.

### Experimental

The title compound, C_20_H_16_N_4_O_2_S_2_, was synthesized by published method (Ünver *et al.*, 2009).

### Refinement

H atoms were positioned geometrically, with C—H = 0.93 and 0.97 Å for aromatic and methylene H, respectively, and constrained to ride on their parent atoms, with *U*_iso_(H) = 1.2Ueq(C). The flip-type disorder means that in their alternative positions, the different types of atoms (in this case sulfur and carbon) occupy positions that are close to each other, which influences their *U^ij^* values. The disordered atoms of the thiophene ring were refined using the following restraints: SIMU, DELU, FLAT and SADI (*SHELXL97*; Sheldrick, 2008).

### Crystal data

**Table:**

C_20_H_16_N_4_O_2_S_2_	*F*(000) = 1696
*M**_r_* = 408.49	*D*_x_ = 1.404 Mg m^−^^3^
Monoclinic, *C*2/*c*	Mo *K*α radiation, λ = 0.71073 Å
Hall symbol: -C 2yc	Cell parameters from 5214 reflections
*a* = 25.287 (3) Å	θ = 1.5–26.6°
*b* = 5.5347 (4) Å	µ = 0.30 mm^−^^1^
*c* = 28.281 (2) Å	*T* = 296 K
β = 102.430 (7)°	Needle, colorless
*V* = 3865.2 (6) Å^3^	0.80 × 0.37 × 0.14 mm
*Z* = 8	

### Data collection

**Table:**

Stoe IPDS II diffractometer	3865 independent reflections
Radiation source: fine-focus sealed tube	2676 reflections with *I* > 2σ(*I*)
graphite	*R*_int_ = 0.037
Detector resolution: 6.67 pixels mm^-1^	θ_max_ = 26.2°, θ_min_ = 1.5°
rotation method scans	*h* = −30→31
Absorption correction: integration (*X-RED32*; Stoe & Cie, 2002)	*k* = −6→6
*T*_min_ = 0.776, *T*_max_ = 0.913	*l* = −34→34
20676 measured reflections	

### Refinement

**Table:**

Refinement on *F*^2^	Primary atom site location: structure-invariant direct methods
Least-squares matrix: full	Secondary atom site location: difference Fourier map
*R*[*F*^2^ > 2σ(*F*^2^)] = 0.049	Hydrogen site location: inferred from neighbouring sites
*wR*(*F*^2^) = 0.141	H-atom parameters constrained
*S* = 1.05	*w* = 1/[σ^2^(*F*_o_^2^) + (0.0757*P*)^2^ + 0.8966*P*] where *P* = (*F*_o_^2^ + 2*F*_c_^2^)/3
3865 reflections	(Δ/σ)_max_ = 0.001
272 parameters	Δρ_max_ = 0.36 e Å^−^^3^
104 restraints	Δρ_min_ = −0.31 e Å^−^^3^

### Special details

**Table:**

Experimental. 281 frames, detector distance = 125 mm
Geometry. All e.s.d.'s (except the e.s.d. in the dihedral angle between two l.s. planes) are estimated using the full covariance matrix. The cell e.s.d.'s are taken into account individually in the estimation of e.s.d.'s in distances, angles and torsion angles; correlations between e.s.d.'s in cell parameters are only used when they are defined by crystal symmetry. An approximate (isotropic) treatment of cell e.s.d.'s is used for estimating e.s.d.'s involving l.s. planes.
Refinement. Refinement of *F*^2^ against ALL reflections. The weighted *R*-factor *wR* and goodness of fit *S* are based on *F*^2^, conventional *R*-factors *R* are based on *F*, with *F* set to zero for negative *F*^2^. The threshold expression of *F*^2^ > σ(*F*^2^) is used only for calculating *R*-factors(gt) *etc*. and is not relevant to the choice of reflections for refinement. *R*-factors based on *F*^2^ are statistically about twice as large as those based on *F*, and *R*- factors based on ALL data will be even larger.

### Fractional atomic coordinates and isotropic or equivalent isotropic displacement parameters (Å^2^)

**Table:**

	*x*	*y*	*z*	*U*_iso_*/*U*_eq_	Occ. (<1)
S1	0.47605 (4)	0.54631 (18)	0.68233 (3)	0.0833 (3)	
O1	0.53985 (8)	0.2178 (4)	0.51294 (7)	0.0694 (6)	
O2	0.67029 (9)	0.1247 (4)	0.51864 (8)	0.0801 (7)	
N1	0.61243 (9)	−0.1968 (5)	0.59495 (8)	0.0610 (6)	
N2	0.59159 (9)	−0.1102 (5)	0.54847 (8)	0.0591 (6)	
N3	0.56138 (8)	0.1266 (4)	0.59653 (7)	0.0550 (5)	
N4	0.53290 (9)	0.2812 (4)	0.61947 (8)	0.0567 (6)	
C1	0.59244 (10)	−0.0525 (5)	0.62262 (9)	0.0571 (7)	
C2	0.56182 (10)	0.0935 (5)	0.54744 (9)	0.0565 (7)	
C3	0.61089 (11)	−0.2103 (5)	0.50850 (10)	0.0595 (7)	
H3A	0.5814	−0.2161	0.4802	0.071*	
H3B	0.6232	−0.3744	0.5163	0.071*	
C4	0.65705 (11)	−0.0615 (5)	0.49698 (9)	0.0558 (6)	
C5	0.68400 (10)	−0.1502 (5)	0.45852 (9)	0.0529 (6)	
C6	0.66851 (13)	−0.3618 (6)	0.43283 (10)	0.0678 (8)	
H6	0.6405	−0.4548	0.4397	0.081*	
C7	0.69452 (16)	−0.4339 (7)	0.39723 (12)	0.0829 (10)	
H7	0.6836	−0.5739	0.3797	0.099*	
C8	0.73677 (16)	−0.2991 (8)	0.38754 (13)	0.0869 (11)	
H8	0.7543	−0.3483	0.3634	0.104*	
C9	0.75304 (14)	−0.0933 (7)	0.41332 (12)	0.0796 (9)	
H9	0.7821	−0.0049	0.4072	0.096*	
C10	0.72666 (12)	−0.0170 (6)	0.44804 (10)	0.0643 (7)	
H10	0.7374	0.1255	0.4648	0.077*	
C11	0.50618 (10)	0.4552 (5)	0.59640 (9)	0.0551 (6)	
H11	0.5061	0.4836	0.5640	0.066*	
C12	0.47593 (11)	0.6068 (5)	0.62279 (9)	0.0572 (7)	
C13	0.44447 (12)	0.8073 (6)	0.60642 (11)	0.0672 (7)	
H13	0.4392	0.8681	0.5751	0.081*	
C14	0.42152 (15)	0.9073 (7)	0.64294 (13)	0.0836 (9)	
H14	0.3991	1.0424	0.6386	0.100*	
C15	0.43555 (16)	0.7853 (7)	0.68508 (13)	0.0888 (11)	
H15	0.4240	0.8288	0.7130	0.107*	
C16	0.60269 (12)	−0.0648 (6)	0.67632 (9)	0.0678 (8)	
H16A	0.6191	−0.2192	0.6870	0.081*	
H16B	0.5684	−0.0557	0.6863	0.081*	
C17	0.63885 (11)	0.1337 (6)	0.70048 (8)	0.0683 (7)	
S2A	0.63477 (7)	0.2526 (3)	0.75375 (5)	0.1001 (6)	0.710 (4)
C18A	0.6783 (3)	0.2503 (12)	0.6854 (3)	0.0779 (12)	0.710 (4)
H18A	0.6878	0.2147	0.6562	0.094*	0.710 (4)
S2B	0.68795 (15)	0.2524 (8)	0.67823 (15)	0.0812 (11)	0.290 (4)
C18B	0.6385 (3)	0.2512 (14)	0.7420 (3)	0.0910 (15)	0.290 (4)
H18B	0.6138	0.2169	0.7612	0.109*	0.290 (4)
C19	0.70600 (12)	0.4412 (6)	0.71870 (13)	0.0940 (9)	
H19	0.7347	0.5382	0.7146	0.113*	
C20	0.68156 (14)	0.4441 (6)	0.75479 (11)	0.0973 (9)	
H20	0.6912	0.5538	0.7801	0.117*	

### Atomic displacement parameters (Å^2^)

**Table:**

	*U*^11^	*U*^22^	*U*^33^	*U*^12^	*U*^13^	*U*^23^
S1	0.1088 (7)	0.0827 (6)	0.0612 (4)	0.0136 (5)	0.0245 (4)	0.0073 (4)
O1	0.0786 (13)	0.0740 (14)	0.0515 (10)	0.0032 (11)	0.0049 (9)	0.0091 (10)
O2	0.0878 (15)	0.0773 (15)	0.0819 (14)	−0.0314 (12)	0.0330 (11)	−0.0300 (12)
N1	0.0583 (13)	0.0669 (15)	0.0587 (13)	−0.0020 (12)	0.0145 (10)	0.0141 (11)
N2	0.0570 (12)	0.0669 (16)	0.0543 (12)	−0.0012 (12)	0.0137 (10)	0.0077 (11)
N3	0.0526 (12)	0.0623 (14)	0.0506 (11)	0.0002 (11)	0.0121 (9)	0.0091 (10)
N4	0.0554 (12)	0.0611 (14)	0.0540 (12)	−0.0021 (11)	0.0126 (10)	0.0055 (11)
C1	0.0520 (14)	0.0639 (17)	0.0562 (14)	−0.0048 (13)	0.0134 (11)	0.0122 (13)
C2	0.0504 (14)	0.0640 (18)	0.0543 (14)	−0.0087 (13)	0.0094 (11)	0.0054 (13)
C3	0.0612 (16)	0.0599 (17)	0.0571 (14)	−0.0074 (13)	0.0119 (12)	−0.0024 (12)
C4	0.0573 (15)	0.0559 (17)	0.0522 (13)	−0.0077 (13)	0.0072 (11)	−0.0023 (12)
C5	0.0546 (14)	0.0545 (16)	0.0471 (12)	0.0000 (12)	0.0053 (10)	0.0021 (11)
C6	0.0774 (19)	0.0589 (18)	0.0657 (16)	−0.0015 (15)	0.0125 (14)	−0.0050 (14)
C7	0.108 (3)	0.067 (2)	0.0728 (19)	0.011 (2)	0.0178 (18)	−0.0160 (16)
C8	0.100 (3)	0.094 (3)	0.074 (2)	0.024 (2)	0.0341 (19)	−0.0010 (19)
C9	0.077 (2)	0.095 (3)	0.0724 (19)	−0.0003 (19)	0.0278 (16)	0.0074 (19)
C10	0.0690 (17)	0.0680 (19)	0.0562 (15)	−0.0079 (15)	0.0138 (13)	−0.0014 (13)
C11	0.0527 (14)	0.0605 (17)	0.0503 (13)	−0.0115 (13)	0.0075 (11)	0.0025 (12)
C12	0.0587 (15)	0.0571 (17)	0.0533 (14)	−0.0084 (13)	0.0064 (11)	0.0009 (12)
C13	0.0761 (18)	0.0598 (17)	0.0629 (15)	−0.0033 (15)	0.0093 (13)	−0.0005 (13)
C14	0.093 (2)	0.068 (2)	0.087 (2)	0.0115 (17)	0.0130 (17)	−0.0029 (17)
C15	0.109 (3)	0.086 (3)	0.077 (2)	0.009 (2)	0.0300 (19)	−0.0089 (19)
C16	0.0717 (18)	0.074 (2)	0.0597 (15)	0.0011 (16)	0.0176 (13)	0.0214 (14)
C17	0.0726 (15)	0.0800 (16)	0.0527 (12)	0.0032 (13)	0.0144 (11)	0.0140 (12)
S2A	0.1227 (11)	0.1241 (12)	0.0609 (8)	−0.0224 (9)	0.0364 (7)	−0.0061 (7)
C18A	0.079 (2)	0.092 (2)	0.071 (2)	−0.005 (2)	0.0339 (18)	0.0026 (18)
S2B	0.0736 (18)	0.0932 (19)	0.0825 (18)	−0.0038 (15)	0.0292 (14)	0.0002 (14)
C18B	0.107 (3)	0.104 (3)	0.065 (3)	−0.010 (2)	0.025 (2)	−0.001 (2)
C19	0.0851 (18)	0.0907 (19)	0.1016 (19)	−0.0082 (16)	0.0097 (15)	0.0082 (16)
C20	0.119 (2)	0.097 (2)	0.0682 (16)	−0.0077 (17)	0.0020 (16)	0.0006 (15)

### Geometric parameters (Å, °)

**Table:**

S1—C15	1.685 (4)	C9—H9	0.9300
S1—C12	1.716 (3)	C10—H10	0.9300
O1—C2	1.225 (3)	C11—C12	1.446 (4)
O2—C4	1.209 (3)	C11—H11	0.9300
N1—C1	1.294 (4)	C12—C13	1.386 (4)
N1—N2	1.392 (3)	C13—C14	1.403 (5)
N2—C2	1.352 (4)	C13—H13	0.9300
N2—C3	1.435 (4)	C14—C15	1.349 (5)
N3—N4	1.369 (3)	C14—H14	0.9300
N3—C1	1.376 (4)	C15—H15	0.9300
N3—C2	1.403 (3)	C16—C17	1.497 (5)
N4—C11	1.273 (3)	C16—H16A	0.9700
C1—C16	1.486 (4)	C16—H16B	0.9700
C3—C4	1.520 (4)	C17—C18A	1.333 (8)
C3—H3A	0.9700	C17—C18B	1.344 (9)
C3—H3B	0.9700	C17—S2B	1.645 (4)
C4—C5	1.486 (4)	C17—S2A	1.668 (3)
C5—C6	1.389 (4)	S2A—C20	1.584 (4)
C5—C10	1.390 (4)	C18A—C19	1.487 (10)
C6—C7	1.375 (5)	C18A—H18A	0.9300
C6—H6	0.9300	S2B—C19	1.545 (5)
C7—C8	1.378 (5)	C18B—C20	1.512 (10)
C7—H7	0.9300	C18B—H18B	0.9300
C8—C9	1.367 (5)	C19—C20	1.302 (6)
C8—H8	0.9300	C19—H19	0.9300
C9—C10	1.367 (4)	C20—H20	0.9300
			
C15—S1—C12	91.12 (17)	C13—C12—C11	128.4 (2)
C1—N1—N2	103.9 (2)	C13—C12—S1	111.3 (2)
C2—N2—N1	113.8 (2)	C11—C12—S1	120.2 (2)
C2—N2—C3	125.9 (2)	C12—C13—C14	111.6 (3)
N1—N2—C3	119.1 (2)	C12—C13—H13	124.2
N4—N3—C1	119.6 (2)	C14—C13—H13	124.2
N4—N3—C2	131.9 (2)	C15—C14—C13	112.4 (3)
C1—N3—C2	108.2 (2)	C15—C14—H14	123.8
C11—N4—N3	120.4 (2)	C13—C14—H14	123.8
N1—C1—N3	111.9 (2)	C14—C15—S1	113.5 (3)
N1—C1—C16	125.8 (3)	C14—C15—H15	123.2
N3—C1—C16	122.3 (3)	S1—C15—H15	123.2
O1—C2—N2	129.8 (3)	C1—C16—C17	112.8 (2)
O1—C2—N3	128.0 (3)	C1—C16—H16A	109.0
N2—C2—N3	102.1 (2)	C17—C16—H16A	109.0
N2—C3—C4	111.5 (2)	C1—C16—H16B	109.0
N2—C3—H3A	109.3	C17—C16—H16B	109.0
C4—C3—H3A	109.3	H16A—C16—H16B	107.8
N2—C3—H3B	109.3	C18A—C17—C18B	101.1 (8)
C4—C3—H3B	109.3	C18A—C17—C16	129.4 (4)
H3A—C3—H3B	108.0	C18B—C17—C16	129.4 (5)
O2—C4—C5	122.1 (3)	C18B—C17—S2B	106.8 (4)
O2—C4—C3	119.7 (3)	C16—C17—S2B	123.8 (2)
C5—C4—C3	118.2 (2)	C18A—C17—S2A	107.1 (4)
C6—C5—C10	118.7 (3)	C16—C17—S2A	123.47 (19)
C6—C5—C4	122.6 (3)	S2B—C17—S2A	112.7 (3)
C10—C5—C4	118.7 (2)	C20—S2A—C17	95.14 (19)
C7—C6—C5	120.1 (3)	C17—C18A—C19	115.1 (6)
C7—C6—H6	119.9	C17—C18A—H18A	122.5
C5—C6—H6	119.9	C19—C18A—H18A	122.5
C6—C7—C8	120.1 (3)	C19—S2B—C17	96.5 (2)
C6—C7—H7	119.9	C17—C18B—C20	114.2 (7)
C8—C7—H7	119.9	C17—C18B—H18B	122.9
C9—C8—C7	120.1 (3)	C20—C18B—H18B	122.9
C9—C8—H8	119.9	C20—C19—C18A	105.5 (4)
C7—C8—H8	119.9	C20—C19—S2B	118.3 (2)
C10—C9—C8	120.2 (3)	C20—C19—H19	127.3
C10—C9—H9	119.9	C18A—C19—H19	127.3
C8—C9—H9	119.9	S2B—C19—H19	114.4
C9—C10—C5	120.7 (3)	C19—C20—C18B	104.1 (4)
C9—C10—H10	119.7	C19—C20—S2A	117.1 (2)
C5—C10—H10	119.7	C19—C20—H20	121.4
N4—C11—C12	117.1 (2)	C18B—C20—H20	134.4
N4—C11—H11	121.4	S2A—C20—H20	121.4
C12—C11—H11	121.4		
			
C1—N1—N2—C2	3.8 (3)	C11—C12—C13—C14	−179.8 (3)
C1—N1—N2—C3	172.3 (2)	S1—C12—C13—C14	0.3 (3)
C1—N3—N4—C11	176.5 (2)	C12—C13—C14—C15	0.1 (4)
C2—N3—N4—C11	−11.2 (4)	C13—C14—C15—S1	−0.5 (4)
N2—N1—C1—N3	−2.0 (3)	C12—S1—C15—C14	0.6 (3)
N2—N1—C1—C16	−179.3 (3)	N1—C1—C16—C17	107.3 (3)
N4—N3—C1—N1	173.8 (2)	N3—C1—C16—C17	−69.8 (3)
C2—N3—C1—N1	−0.2 (3)	C1—C16—C17—C18A	−29.9 (4)
N4—N3—C1—C16	−8.8 (4)	C1—C16—C17—C18B	146.2 (2)
C2—N3—C1—C16	177.2 (2)	C1—C16—C17—S2B	−32.7 (4)
N1—N2—C2—O1	177.2 (3)	C1—C16—C17—S2A	149.2 (2)
C3—N2—C2—O1	9.5 (5)	C18A—C17—S2A—C20	0.72 (9)
N1—N2—C2—N3	−3.8 (3)	C18B—C17—S2A—C20	−19 (2)
C3—N2—C2—N3	−171.4 (2)	C16—C17—S2A—C20	−178.5 (3)
N4—N3—C2—O1	8.5 (5)	S2B—C17—S2A—C20	3.2 (2)
C1—N3—C2—O1	−178.6 (3)	C18B—C17—C18A—C19	2.5 (2)
N4—N3—C2—N2	−170.6 (2)	C16—C17—C18A—C19	179.5 (3)
C1—N3—C2—N2	2.4 (3)	S2B—C17—C18A—C19	−158 (2)
C2—N2—C3—C4	72.9 (3)	S2A—C17—C18A—C19	0.31 (12)
N1—N2—C3—C4	−94.1 (3)	C18A—C17—S2B—C19	19 (2)
N2—C3—C4—O2	−4.8 (4)	C18B—C17—S2B—C19	−0.80 (10)
N2—C3—C4—C5	176.1 (2)	C16—C17—S2B—C19	178.3 (3)
O2—C4—C5—C6	−178.9 (3)	S2A—C17—S2B—C19	−3.4 (2)
C3—C4—C5—C6	0.3 (4)	C18A—C17—C18B—C20	−2.7 (2)
O2—C4—C5—C10	1.6 (4)	C16—C17—C18B—C20	−179.6 (3)
C3—C4—C5—C10	−179.3 (2)	S2B—C17—C18B—C20	−0.60 (12)
C10—C5—C6—C7	−1.1 (4)	S2A—C17—C18B—C20	158 (2)
C4—C5—C6—C7	179.3 (3)	C17—C18A—C19—C20	−1.4 (2)
C5—C6—C7—C8	1.3 (5)	C17—C18A—C19—S2B	169.1 (9)
C6—C7—C8—C9	0.1 (5)	C17—S2B—C19—C20	2.3 (2)
C7—C8—C9—C10	−1.6 (5)	C17—S2B—C19—C18A	−8.0 (7)
C8—C9—C10—C5	1.7 (5)	C18A—C19—C20—C18B	−0.31 (17)
C6—C5—C10—C9	−0.3 (4)	S2B—C19—C20—C18B	−2.7 (3)
C4—C5—C10—C9	179.2 (3)	C18A—C19—C20—S2A	2.0 (3)
N3—N4—C11—C12	178.4 (2)	S2B—C19—C20—S2A	−0.4 (2)
N4—C11—C12—C13	179.1 (3)	C17—C18B—C20—C19	2.0 (2)
N4—C11—C12—S1	−1.1 (3)	C17—C18B—C20—S2A	−168.8 (9)
C15—S1—C12—C13	−0.5 (2)	C17—S2A—C20—C19	−1.8 (2)
C15—S1—C12—C11	179.6 (2)	C17—S2A—C20—C18B	8.2 (7)

### Hydrogen-bond geometry (Å, °)

**Table:**

*D*—H···*A*	*D*—H	H···*A*	*D*···*A*	*D*—H···*A*
C11—H11···O1	0.93	2.35	2.981 (3)	125
